# Effect of statin on life prognosis in Japanese patients undergoing hemodialysis

**DOI:** 10.1371/journal.pone.0224111

**Published:** 2019-10-22

**Authors:** Yuki Ota, Mineaki Kitamura, Kumiko Muta, Hiroshi Yamashita, Tadashi Uramatsu, Yoko Obata, Takashi Harada, Satoshi Funakoshi, Hiroshi Mukae, Tomoya Nishino

**Affiliations:** 1 Department of Nephrology, Nagasaki University Hospital, Nagasaki, Japan; 2 Division of Blood Purification, Nagasaki University Hospital, Nagasaki, Japan; 3 Department of Nephrology, Nagasaki Renal Center, Nagasaki, Japan; 4 Department of Respiratory Medicine, Nagasaki University Graduate School of Biomedical Sciences, Nagasaki, Japan; International University of Health and Welfare, School of Medicine, JAPAN

## Abstract

The effect of statin on hemodialysis patients is controversial. Although previous large-scale studies did not clarify its effect in this population, recent studies suggest that statins could be useful in reducing the risk of cardiovascular events and all-cause mortality in specific groups of patients undergoing hemodialysis. The aforementioned large-scale studies included a small percentage of Asians, and few studies have investigated the effects of statins in Asians undergoing hemodialysis. Thus, we investigated the benefits of statins in patients undergoing maintenance hemodialysis at a single center in Japan. We obtained demographic, clinical, and hemodialysis data of all patients who underwent maintenance hemodialysis at the Nagasaki Renal Center between July 2011 and June 2012. Patients were followed-up until June 2018. We studied 339 patients, of which 51 (15.0%) were prescribed pitavastatin. The mean observation period was 4.1±2.3 years, 43% were women, and the median hemodialysis vintage at baseline was 4.7 years. During the follow-up, 198 patients (58%) died, of which 22 (43%) were prescribed pitavastatin and 176 (61%) were not prescribed any statins. After propensity score matching based on age, sex, dialysis vintage, dialysis time, diabetes mellitus, ischemic heart disease, dry weight, left ventricular ejection fraction, and serum albumin, an intergroup comparison between those who received statins and those who did not (44 patients in each group) showed significant differences in survival rate based on the log-rank test (P<0.05). Although the causes of death did not differ significantly between groups, deaths due to cardiovascular events, infections, and cancer were fewer in the group prescribed statins. Our results suggest that statins may reduce mortality in Japanese patients undergoing maintenance hemodialysis. Although potential residual confounders exist, statins may have an influence on the reduction in the incidence of cardiovascular events, infections, and cancer. Nevertheless, further studies are required to prove this hypothesis.

## Introduction

Patients on hemodialysis have a greater risk of developing cardiovascular events, which are associated with life prognosis [1,2]. Anemia correction and smoking cessation, blood pressure and diabetes management, calcium and phosphorus management, and use of beta blockers have been associated with decreasing cardiovascular events and reduction in mortality risk in dialysis patients [3–8]. Moreover, statins are useful for primary and secondary prevention of cardiovascular events by lowering low-density lipoprotein (LDL) cholesterol levels [9–12]; however, the favorable effect of statin was not observed in two large-scale clinical trials of patients on hemodialysis. A previous study (4D study) assessed the risk of developing a combined primary endpoint, i.e., cardiovascular disease death, nonfatal myocardial infarction, and nonfatal stroke, in patients on hemodialysis. Although an 8% risk reduction in the atorvastatin group was shown, no significant difference between the atorvastatin and placebo groups was noted [13]. Moreover, the risk of all-cause death, cardiovascular events, and cerebrovascular disease was almost the same in the two groups [13]. In the AURORA study, the same endpoint as that in the 4D study was set in the rosuvastatin and placebo groups. Although the LDL cholesterol level decreased in the rosuvastatin group, the risk of cardiovascular events was not reduced [14]. Thus, based on the results of large clinical studies and according to the Kidney Disease: Improving Global Outcomes clinical practice guideline for lipid management in CKD, starting statins in patients on dialysis is not recommended [15].

Dialysis patients in Japan have a relatively high life expectancy compared with those in Europe and the United States [16,17]. In addition, kidney transplantation, especially deceased-donor kidney transplantation, is not popular in Japan and, thus, the mean dialysis vintage in Japan tends to be higher than that in other countries [18]. These factors make a long-term observational study of hemodialysis patients feasible in Japan; however, few studies on the effect of statin on Japanese patients undergoing hemodialysis have been conducted [16,17]. Thus, this study aimed to retrospectively examine the effects of statins on the life prognosis of patients undergoing hemodialysis at a single center in Japan.

## Materials and methods

### Patients

This was a retrospective observational study at a hemodialysis center. The subjects were patients who underwent hemodialysis at the Nagasaki Renal Center from July 2011 to June 2012. The inclusion criteria and exclusion criteria were similar to those in a previous report [19]. Briefly, patients who were at least 20 years of age and with a dialysis vintage of at least 90 days were included, and those who were up to 19 years of age, had a dialysis vintage of <90 days, left the Nagasaki Renal Center during the observation period, or died were excluded. The observation period was from July 2011 to June 2018.

### Data collection

Patients' background data, including age, sex, history of dialysis, dialysis conditions, blood examinations, chest radiographs, echocardiography results, and information on pre-existing conditions, complications, and medications were obtained from medical records. These data were obtained from the results of examinations which were performed in the patient's birth month. A ratio of 1:200 was applied to convert the darbepoetin alfa and epoetin beta pegol doses to their equivalent epoetin doses [20].

### Statistical analyses

Categorical variables are expressed as number (%) and continuous variables, as mean±standard deviations. Non-normally distributed data are presented as median values with interquartile ranges. Statistical analyses were performed using JMP 14 software (SAS Institute Inc., Cary, NC). The analyses included a comparison between two groups: statin and non-statin groups. Continuous variables were compared using the Mann-Whitney U-test, while categorical variables were assessed by the Chi-square test. Univariate and multivariate Cox proportional-hazards model analyses were also performed. Model 1 was adjusted for age and sex only; model 2, for age, sex, dialysis vintage, dialysis time, diabetes mellitus, ischemic heart disease, cerebral infarction, systolic blood pressure, body mass index, left ventricular ejection fraction, and serum albumin; and model 3, for age, sex, dialysis vintage, dialysis time, diabetes mellitus, cerebral infarction, systolic blood pressure, body mass index, left ventricular ejection fraction, hemoglobin, serum albumin, serum corrected calcium, serum phosphate, intact-parathyroid hormone, serum creatinine, total cholesterol, and drugs (erythropoiesis-stimulating agent, phosphate binders, cinacalcet, and vitamin D).

Moreover, propensity score matching with a Caliper coefficient set at 0.2 was performed, and survival time analysis was conducted by the log-rank test. To estimate the propensity score, age, sex, dialysis vintage, dialysis time, diabetes history, ischemic heart disease history, cerebral infarction history, body weight, ejection fraction, and serum albumin were selected as parameters because of their clinical importance and data availability. In addition, we estimated another propensity score using age, sex, dialysis vintage, dialysis time, diabetes history, ischemic heart disease history, cerebral infarction history, body mass index, ejection fraction, serum albumin, and HbA1c. The missing data were removed from the analyses and only the remaining data were used.

This study was approved by the ethics committee of the Nagasaki Renal Center (Nagasaki, Japan) (30001) and performed according to the 1964 Declaration of Helsinki and its later amendments. Although the patients in this study were informed, consent was not obtained as a medical records-based retrospective analysis was conducted, and the included patients were anonymized. The ethics committee approved the waiver of consent.

## Results

This study included 339 patients, and 43% were women. The mean follow-up period was 4.1±2.3 years, mean age was 67.3±13.3 years, median dialysis vintage was 4.7 years, and median dialysis time was 4 h. The prevalence of ischemic heart disease was 34.2% and that of diabetes was 34.5%. Fifty-one patients (15.0%) were prescribed statin (statin group); only pitavastatin was available in the Nagasaki Renal Center. The dose of pitavastatin was 1 mg in 46 patients and 2 mg in 5 patients. In the statin group, statin was continuously prescribed until the last follow-up assessment in almost all cases; however, the total administration period could not be confirmed. During the observation period, 198 patients (58%) died: 22 (43%) in the statin group and 176 (61%) in the non-statin group (Table 1).

**Table 1:** Baseline characteristics of the statin and non-statin groups.

	All (N = 339)	Statin group (N = 51)	Non-statin group (N = 288)	P value
Death (%)	58.4	43.1	61.1	0.02
Age (years)	67.3±13.3	65.9±11.7	67.6±13.6	0.39
Female (%)	42.8	64.7	38.9	<0.001
Dialysis vintage^a^ (years)	4.7 (1.9–10.2)	2.9 (1.1–10.3)	5.0 (2.0–10.2)	0.34
Dialysis time^a^ (h)	4 (3–4)	4 (3–4)	4 (3–4)	0.51
Ischemic heart disease (%)	34.2	49.0	31.6	0.02
Diabetes mellitus (%)	34.5	52.9	31.3	0.004
Cerebral hemorrhage (%)	24.8	21.8	25.4	0.73
Cerebral infarction (%)	6.5	5.9	6.6	0.57
Arteriosclerosis obliterans (%)	16.8	17.7	16.7	0.84
Cardiothoracic ratio (%)	52.2±5.8	51.9±4.4	52.2±6.0	0.72
Body weight (kg)	52.1±11.0	49.6±9.8	52.4±11.2	0.09
Body mass index (kg/m^2^)	20.6±3.5	20.4±2.9	20.6±3.6	0.69
Systolic blood pressure (mmHg)	150±24.4	155±23.4	149±24.5	0.08
Diastolic blood pressure (mmHg)	78.0±13.6	79.6±13.3	77.7±13.7	0.37
Left ventricular ejection fraction (%)	64.8±10.2	67.9±9.8	64.3±10.2	0.02
Hb (g/dL)	10.8±1.4	11.0±1.1	10.8±1.4	0.23
Ferritin^a^ (ng/mL)	53.3 (21.4–154.2)	63.1 (14.1–133.4)	51.0 (21.7–162.4)	0.36
TSAT (%)	23.9±13.3	25.5±11.3	23.9±13.3	0.48
Alb (g/dL)	3.6±0.4	3.8±0.3	3.5±0.4	<0.001
cCa (mg/dL)	9.2±0.8	9.1±0.6	9.3±0.8	0.33
P (mg/dL)	5.6±1.6	5.8±1.3	5.6±1.7	0.46
Intact-PTH^a^ (pg/mL)	72 (28–154)	37 (19–136)	79 (30.5–157.5)	0.39
ALP (IU/L)	282±133	257±106	286±136	0.14
BUN (mg/dL)	68.1±18.2	70.2±13.9	67.7±18.8	0.36
Cr (mg/dL)	10.2±3.4	10.5±3.0	10.2±3.5	0.61
Total cholesterol (mg/dL)	162±37.1	158±31.2	162±38.1	0.50
Triglycerides (mg/dL)	105.6±63.5	109±66.6	105±63.0	0.70
CRP^a^ (mg/dL)	0.18 (0.07–0.53)	0.07 (0.05–0.19)	0.21 (0.08–0.64)	0.04
HbA1c (%)	5.4±1.1	6.0±1.5	5.3±1.0	<0.001
Anti-platelet drugs (%)	39.2	49.0	37.5	0.12
Warfarin (%)	7.1	5.9	7.3	0.50
ESA^a^ (IU/week)	4500 (2000–8000)	4000 (2000–8000)	4500 (2000–8000)	0.47
Iron (%)	31.0	19.6	19.1	0.62
[Phosphate binders] (%)	65.2	74.5	63.5	0.13
Calcium carbonate (%)	47.5	56.9	45.8	0.17
Lanthanum carbonate (%)	31.0	35.3	30.2	0.51
Sevelamer (%)	2.9	1.96	3.15	0.53
Cinacalcet (%)	16.5	13.7	17.1	0.68
Vitamin D (%)	66.1	74.5	65.5	0.26
Oral antidiabetic drug (%)	12.6	3.5	9.1	0.02
Insulin (%)	10.3	3.0	7.3	0.03

Continuous variables are shown as mean ± standard deviation and categorical variables as percentage or number (percentage).

Hb: hemoglobin, TSAT: transferrin saturation, Alb: albumin, cCa: corrected calcium, P: phosphate, PTH: parathyroid hormone, ALP: alkali-phosphatase, BUN: blood urea nitrogen, Cr: creatinine, CRP: C-reactive protein, HbA1c: hemoglobin A1c, ESA: erythropoiesis-stimulating agent

^a^Median (interquartile range)

The proportion of women and the number of patients with ischemic heart disease and diabetes were significantly higher in the statin group than in the non-statin group (Table 1). Left ventricular ejection fraction and serum albumin levels were also significantly higher in the statin group (Table 1).

Univariate Cox proportional-hazards analysis is shown in Table 2. Univariate and multivariate Cox proportional-hazards analyses showed that statin administration has a significant influence on the risk of death. For example, in model 3, statin significantly decreased the risk of mortality [hazard ratio (HR) 0.56, 95% confidence interval (CI) 0.48–0.96; P = 0.02] (Table 2).

**Table 2:** Cox regression analysis.

Variables	Univariate			Model 1			Model 2			Model 3		
	HR	95% CI	P value	HR	95% CI	P value	HR	95% CI	P value	HR	95% CI	P value
Age /year	1.07	1.05–1.08	<0.001	1.04	1.03–1.05	<0.001	1.04	1.02–1.05	<0.001	1.03	1.02–1.05	<0.001
Female	0.93	0.70–1.24	0.63	0.91	0.73–1.13	0.42	0.74	0.56–0.95	0.35	0.82	0.53–0.95	0.30
Dialysis vintage /year	0.96	0.94–0.98	<0.001				1.01	0.98–1.03	0.63	1.01	0.99–1.04	0.32
Dialysis time /hour	0.33	0.25–0.44	<0.001				0.60	0.43–0.84	0.003	0.58	0.41–0.82	0.002
Ischemic heart disease history	1.17	0.87–1.56	0.28				1.14	0.84–1.54	0.40	1.01	0.63–1.06	0.95
Diabetes mellitus history	1.58	1.19–2.09	0.002				1.65	1.20–2.25	0.002	1.51	0.89–1.56	0.02
Cerebral hemorrhage history	0.90	0.48–1.54	0.72									
Cerebral infarction history	2.05	1.51–2.75	<0.001				1.37	1.00–1.85	0.04	1.31	0.96–1.67	0.10
Arteriosclerosis obliterans history	1.36	0.95–1.91	0.08									
Cardiothoracic ratio /1%	1.09	1.06–1.11	<0.001									
Dry weight /kg	0.96	0.95–0.97	<0.001									
Body mass index	0.88	0.84–0.92	<0.001				0.93	0.88–0.98	0.008	0.94	0.89–0.99	0.03
sBP /10 mmHg	0.98	0.93–1.03	0.41				0.97	0.91–1.00	0.07	0.96	0.90–1.01	0.12
dBP /10 mmHg	0.75	0.68–0.83	<0.001									
LVEF / 1%	0.97	0.96–0.98	<0.001							0.98	0.96–0.99	<0.001
Hb/g/dL	0.83	0.75–0.93	0.001							1.03	0.91–1.18	0.65
Ferritin /10 ng/mL	1.01	1.00–1.02	0.004									
TSAT /1%	1.17	0.31–4.13	0.81									
Alb /g/dL	0.19	0.14–0.26	<0.001				0.35	0.24–0.50	<0.001	0.69	0.48–0.99	<0.001
cCa /mg/dL	1.12	0.92–1.36	0.24							1.22	0.98–1.52	0.07
P /mg/dL	0.83	0.88–1.01	0.11							1.11	1.00–1.24	0.04
Intact-PTH /10 pg/mL	0.98	0.96–0.99	<0.001							1.00	0.98–1.01	0.80
ALP /10 IU/L	1.01	1.00–1.02	0.03									
BUN /10 mg/dL	0.86	0.79–0.94	<0.001									
Cr / mg/dL	0.81	0.78–0.85	<0.001							0.89	0.83–0.95	<0.001
T-chol /10 mg/dL	0.94	0.90–0.98	<0.001							0.99	0.95–1.03	0.63
Triglyceride /10 mg/dL	0.97	0.95–1.00	0.03									
CRP /mg/dL	1.07	1.01–1.13	0.03									
Anti-platelet drugs	1.15	0.86–1.52	0.33									
Warfarin	1.26	0.73–2.04	0.37									
ESA /100 U/week	1.00	1.00–1.01	0.001							1.00	1.00–1.00	0.92
Iron use	0.97	0.67–1.37	0.88									
Phosphate binders use	0.35	0.27–0.47	<0.001							1.04	0.72–1.52	0.83
Calcium carbonate use	0.54	0.41–0.72	<0.001									
Lanthanum carbonate use	0.42	0.29–0.58	<0.001									
Sevelamer use	0.11	0.01–0.48	0.03									
Cinacalcet use	0.43	0.26–0.67	<0.001							1.12	0.64–1.91	0.66
Vitamin D use	0.59	0.44–0.78	<0.001							0.83	0.60–1.16	0.28
Statin use	0.55	0.34–0.84	0.01	0.60	0.37–0.92	0.02	0.67	0.47–0.92	0.02	0.56	0.48–0.96	0.02

Model 1 was adjusted for age and sex; model 2, for age, sex, dialysis vintage, dialysis time, diabetes mellitus, ischemic heart disease, cerebral infarction, body mass index, left ventricular ejection fraction, and serum albumin; model 3, for age, sex, dialysis vintage, dialysis time, diabetes mellitus, cerebral infarction, body mass index, left ventricular ejection fraction, hemoglobin, serum albumin, serum corrected calcium, serum phosphate, intact parathyroid hormone, serum creatinine, total cholesterol, and drugs (erythropoiesis-stimulating agent, phosphate binders, cinacalcet, and vitamin D). sBP: systolic blood pressure, dBP: diastolic blood pressure, LVEF: left ventricular ejection fraction, Hb: hemoglobin, TSAT: transferrin saturation, Alb: albumin, cCa: corrected calcium, P: phosphate, PTH: parathyroid hormone, ALP: alkali-phosphatase, BUN: blood urea nitrogen, Cr: creatinine, T-chol: total cholesterol, CRP: C-reactive protein, ESA: erythropoiesis-stimulating agent

**Fig 1:**
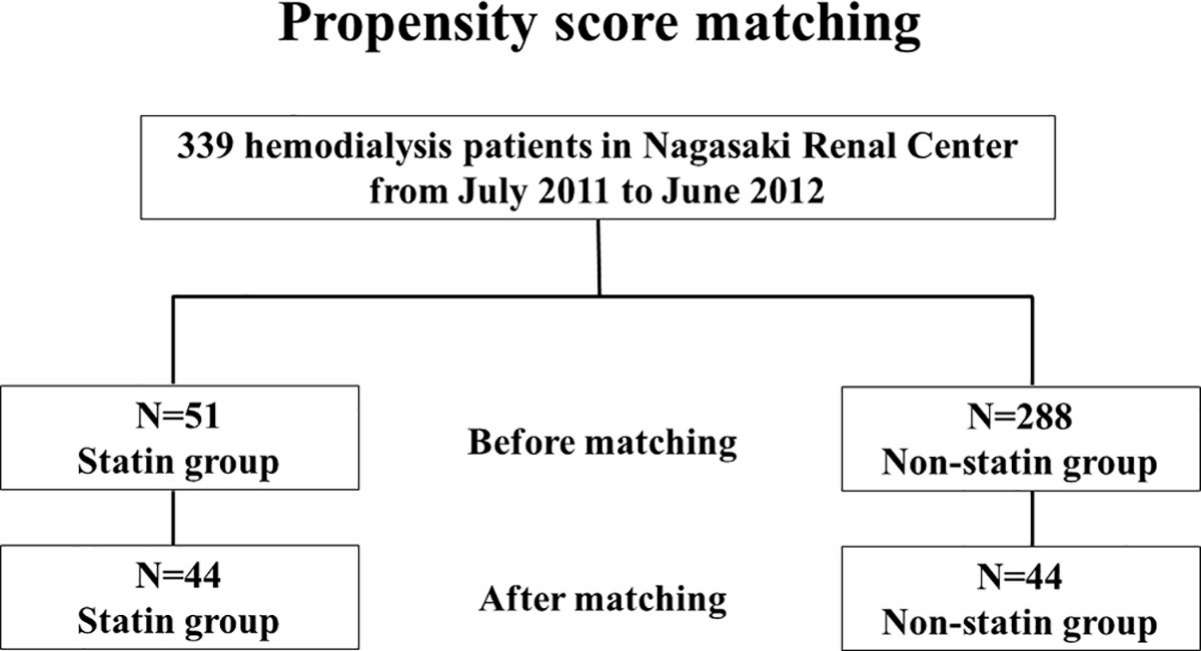
Flowchart of propensity score matching.

Subsequently, we performed propensity score matching and found no significant difference between the statin and non-statin groups in terms of the baseline characteristics that were used to estimate the propensity score (Table 3). Regarding the total cholesterol level, which was not used to estimate the propensity score, there was no difference between the statin group and the non-statin group (statin group: 159±33 mg/dL, non-statin group: 166±41 mg/dL; P = 0.64). Survival analysis was also performed (44 patients in each group), and significant differences in survival rate between the groups were observed (Fig 1).

**Table 3:** Characteristics of the propensity score matched cohort.

	Statin group (N = 44)	Non-stain group (N = 44)	P value
Age (years)	65.3±11.9	66.6±13.7	0.64
Female (%)	61.3	56.8	0.83
Dialysis vintage^a^ (year)	3.4 (1.0–12.2)	5.4 (2.9–10.1)	0.70
Dialysis time^a^ (h)	4 (3–4)	4 (3–4)	0.85
Ischemic heart disease (%)	45.5	50.0	0.83
Diabetes mellitus (%)	45.5	38.6	0.67
Cerebral infarction (%)	20.5	25.0	0.80
Dry weight (kg)	50.2±9.8	47.7±9.7	0.22
Left ventricular ejection fraction (%)	67.0±9.8	69.3±8.4	0.24
Alb (g/dL)	3.7±0.3	3.7±0.3	0.73

Continuous variables are shown as mean ± standard deviation and categorical variables as percentage or number (percentage). Alb: albumin

^a^Median (interquartile range)

**Fig 2:**
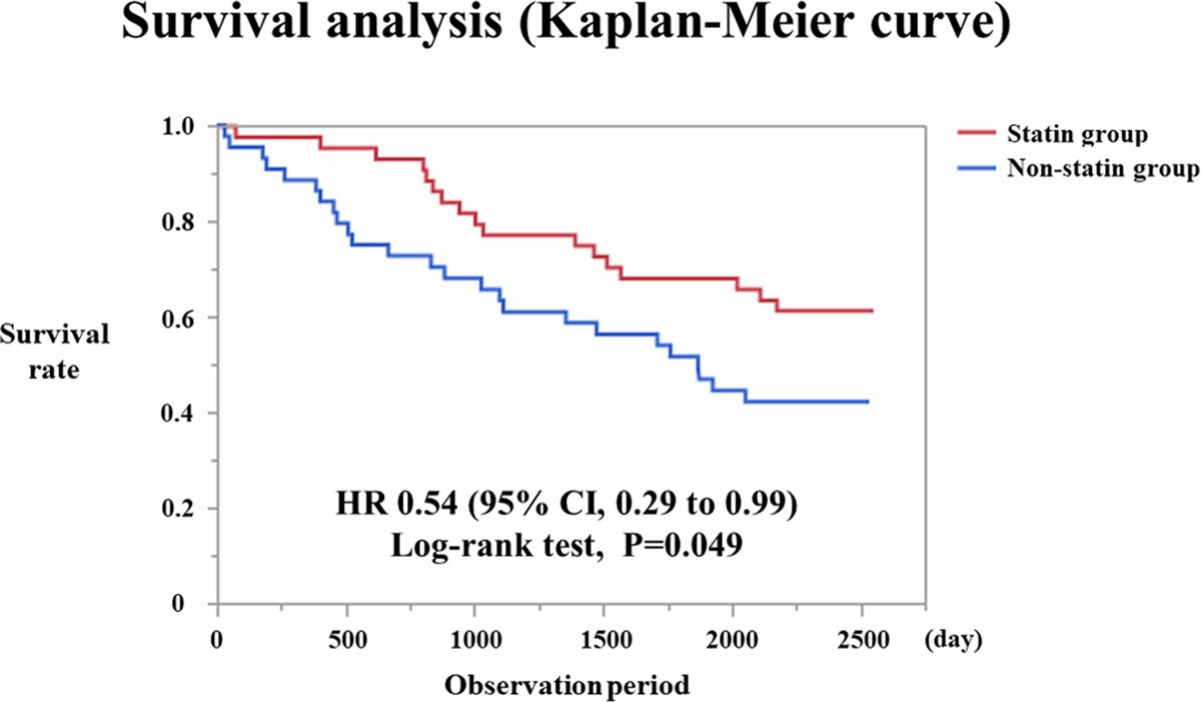
Survival analysis (Kaplan-Meier curve).

Based on the survival analysis, the statin group had a significantly lower risk of death than the non-statin group (HR 0.54, 95% CI 0.29–0.99; log-rank test P = 0.049) (Fig 2).

The number of patients who had cardiac death and malignancy were higher in the non-statin group, whereas, the number of patients who died of stroke was higher in the statin group; however, no significant differences were observed (Table 4).

**Table 4:** Causes of death.

	Statin group (N = 44)	Non-statin group (N = 44)	P value
[Death]	17	25	
[Cardiac death]	5	10	0.26
Myocardial infarction	2	1	0.62
Heart failure	2	3	1.0
Fatal arrhythmia	0	1	1.0
Sudden death	1	5	0.20
[Infection]	9	11	0.79
Sepsis	4	3	1.0
Pneumonia	4	5	0.74
Others	1	3	1.0
[Cancer]	0	4	0.12
[Stroke]	3	0	0.24
Cerebral infarction	2	0	0.49
Cerebral hemorrhage	1	0	1.0

We performed another propensity score matching analysis, which included HbA1c and body mass index instead of dry weight in addition to parameters of the former propensity score matching. There were 31 patients in each group and the results showed that the statin group had a significantly lower risk of death than the non-statin group (HR 0.50, 95% CI 0.24–0.98; log-rank test P = 0.041) (S1 Fig. and S1 Table). There was no significant difference in the total cholesterol levels between the statin group and the non-statin group, similar to the result of the former propensity score matching analysis (data not shown).

## Discussion

This study investigated the efficacy of statins in Japanese patients on dialysis. Statin was used in 15% of 339 eligible patients. Univariate and multivariate Cox regression analyses and the survival analysis between the propensity score matched groups showed that pitavastatin significantly decreased the mortality risk in hemodialysis patients.

Although the 4D study and AURORA study suggested that statins are less effective in hemodialysis patients, the proportions of Asian patients included in these studies were small [13,14]; thus, the results could not be generalized. Racial differences with respect to the effect of statins remain to be fully elucidated [21–23]; nevertheless, a retrospective study in Asians suggested that statins may improve the survival rate after acute myocardial infarction in patients on dialysis [24]. A systematic review and meta-analysis demonstrated that statins reduce the risk of cardiovascular events in patients with CKD, including those on hemodialysis [25]. Furthermore, the long-term follow-up of the 4D study showed a significant difference in the onset of cardiovascular events, according to the arms (statin or placebo) of the original 4D study, suggesting a legacy effect of statins in hemodialysis patients [26].

Apart from lowering LDL cholesterol, statins have pleiotropic effects, including the expression of nitric oxide in the vascular endothelium, stabilization of atherosclerotic plaques, and suppression of proinflammatory cytokines and reactive oxygen species [27–31]. Statins may also be effective in reducing the reactivity of the heart muscle and suppressing the development of myocardial hypertrophy and fibrosis [32]. With regard to the anti-inflammatory effect, statins may clinically reduce the incidence of pneumonia, malignancy, dementia, venous thrombosis, osteoporosis, and pancreatitis [33–38]. In our study, no significant tendency in the cause of death between the statin and non-statin groups was observed. This could be because the number of participants was small; thus, a larger scale study to elucidate the effect of statins on the incidence of infection in hemodialysis patients is warranted. Moreover, the mechanism underlying the improvement seen in life prognosis with statin use is difficult to establish based on our study’s results, and thus, further studies will be required in this regard.

This study has several limitations that should be taken into account while interpreting the results. This was a single-institution retrospective study. The number of patients included was small; the number of patients for propensity score matching was significantly smaller. Moreover, LDL cholesterol was not measured in all patients; thus, the level of LDL cholesterol was not assessed in this study. Other unknown unmeasured items could not be adjusted between the two groups. In addition, the statin group tended to have numerous comorbidities, such as ischemic heart disease and diabetes. Hence, follow-up consultations with specialists outside the dialysis facility were performed more frequently in the statin group. Moreover, the time of initiation of statins was not available and the duration of statin prescription was not well documented. Statins might have been prescribed before the initiation of hemodialysis in some cases, and other statins, such as atorvastatin, may have been prescribed before admission to the Nagasaki Renal Center.

Nonetheless, this study has several strengths. As the dialysis vintage of hemodialysis patients in Japan is relatively longer than that of patients in other countries, our study was able to perform a long-term observation. Only pitavastatin was used in this study because of its availability in the facility. This helped to preclude any confounding bias that could have arisen due to the use of different statins with different inherent characteristics. Moreover, the causes of death data could be obtained in detail, and almost all patients could be followed up during the observation period.

## Conclusion

This study showed that pitavastatin could reduce mortality of hemodialysis patients. Although the effect of statin in hemodialysis patients has remained controversial, long-term statin administration could decrease the mortality risk in these patients. Nonetheless, no significant difference in the cause of death regardless of the use of statins was noted in this study. Moreover, the factors that could influence the effect of statins on survival prognosis remain to be further clarified.

## Supporting information

S1 Data Patients’ data are supplied in a supporting information file. (XLSX)

S1 Fig Survival analysis (Kaplan-Meier curve) of the matched cohort. The propensity score was estimated using age, sex, dialysis vintage, dialysis time, diabetes history, ischemic heart disease history, cerebral infarction history, body mass index, ejection fraction, serum albumin, and hemoglobin A1c.
(TIF)

S1 Table Characteristics of the propensity score matched cohort. The propensity score was estimated using age, sex, dialysis vintage, dialysis time, diabetes history, ischemic heart disease history, cerebral infarction history, body mass index, ejection fraction, serum albumin, and hemoglobin A1c.
(DOCX)
