# Correction: Effect of statin on life prognosis in Japanese patients undergoing hemodialysis

**DOI:** 10.1371/journal.pone.0228298

**Published:** 2020-01-21

**Authors:** Yuki Ota, Mineaki Kitamura, Kumiko Muta, Hiroshi Yamashita, Tadashi Uramatsu, Yoko Obata, Takashi Harada, Satoshi Funakoshi, Hiroshi Mukae, Tomoya Nishino

There is an error in affiliation 1 for authors Yuki Ota, Mineaki Kitamura, Kumiko Muta, Hiroshi Yamashita, Tadashi Uramatsu, Yoko Obata, and Tomoya Nishino. The correct affiliation 1 is: Department of Nephrology, Nagasaki University Graduate School of Biomedical Sciences, Nagasaki, Japan.
