# Supplementary material for: Effect of statin on life prognosis in Japanese patients undergoing hemodialysis
Source: PLoS One. 2019 Oct 22;14(10):e0224111. doi: 10.1371/journal.pone.0224111 (PMC6804988; doi:10.1371/journal.pone.0224111)
Supplement: S1 Table — The propensity score was estimated using age, sex, dialysis vintage, dialysis time, diabetes history, ischemic heart disease history, cerebral infarction history, body mass index, ejection fraction, serum albumin, and hemoglobin A1c. (DOCX) [file pone.0224111.s003.docx]

|  | Statin group  (N=31) | Non-stain group  (N=31) | P value |
| --- | --- | --- | --- |
| Age (years) | 65.5±9.6 | 68.2±11.4 | 0.31 |
| Female (%) | 54.8 | 54.8 | 1.00 |
| Dialysis vintage^a^ (year) | 2.3 (0.7–7.7) | 3.9 (1.2–7.3) | 0.53 |
| Dialysis time^a^ (h) | 4 (3–4) | 3.5 (3–4) | 0.13 |
| Ischemic heart disease (%) | 48.4 | 55.9 | 0.31 |
| Diabetes mellitus (%) | 67.7 | 67.7 | 1.00 |
| Cerebral infarction (%) | 22.6 | 25.8 | 0.77 |
| BMI | 21.1±2.8 | 21.3±4.2 | 0.96 |
| Left ventricular ejection fraction (%) | 66.9±10.5 | 65.5±8.2 | 0.30 |
| Alb (g/dL) | 3.7±0.3 | 3.7±0.4 | 0.91 |
| HbA1c | 5.7±1.4 | 5.8±1.2 | 0.63 |

**S1 Table 1. Characteristics of the propensity score matched cohort**
